# Exploring the efficacy of drought tolerant, IAA-producing plant growth-promoting rhizobacteria for sustainable agriculture

**DOI:** 10.1080/15592324.2025.2452331

**Published:** 2025-01-15

**Authors:** Malika Uzma, Atif Nisar, Atia Iqbal, Shahida Hasnain, Mohamed H. Mahmoud, Muhammad Abdul Rahim, Tehseen Gull, Roberto Castro-Muñoz, Eliasse Zongo

**Affiliations:** a Department of Medical Lab Technology, Times Institute, Multan, Pakistan; b Department of Pathobiology, Faculty of Veterinary Sciences, Bahauddin Zakariya University, Multan, Pakistan; c Department of Microbiology and Molecular Genetics, Matital campus, The Women University Multan, Multan, Pakistan; d Institute of Microbiology and Molecular Genetics, Quaid-e-Azam Campus, University of the Punjab, Lahore, Pakistan; e Department of Biochemistry, College of Science, King Saud University, Riyadh, Saudi Arabia; f Department of Food Science & Nutrition, Faculty of Medicine and Allied Health Sciences, Times Institute, Multan, Pakistan; g Department of Chemistry, Times Institute, Multan, Pakistan; h Department of Sanitary Engineering, Faculty of Civil and Environmental Engineering, Gdansk University of Technology, Gdansk, Poland; i Laboratory of Research and Teaching in Animal Health and Biotechnology, Bobo-Dioulasso, Burkina Faso

**Keywords:** Isolation, characterization, zinc solubility, motility, hydrophobicity, biofilm

## Abstract

The growing human population and abiotic stresses pose significant threats to food security, with PGPR favorable as biofertilizers for plant growth and stress relief. In one study, soil samples from both cultivated and uncultivated plants in various cities were used to isolate rhizobacterial populations. Using 50 soil samples from both cultivated and uncultivated plants, isolated rhizobacterial populations were screened for various biochemical changes, PGP activities and morphological characteristics. A total of 199 rhizobacteria were isolated and screened for IAA production. The strain M28 produced maximum IAA 378.44 ± 2.5 µg ml^−1^, M9 formed only 34.72 ± 0.15 µg ml^−1^. About 19% of IAA producers were isolated from Multan, 18% Lahore, 15% from soils of Faisalabad and Sheikhupura, while 7% from Gujrat. The 21 isolates were drought tolerant to −0.14Mpa, 14 of those were PSB and 15 were N fixers. In PGP traits, maximum zinc solubility was expressed by M4 as 2 ± 0.5 cm of zone. The strain M22 produced amount of HCN, 40.12 ± 0.052 ppm. All isolates showed diverse behavior in biocompatibility, motility patterns and hydrophobicity. Selected drought tolerant strains were genetically identified by ribotyping. Multitrait PGPR could be effective biofertilizers rather than with single trait. The strain M28 having highest production of IAA, was gelatinase, methyl red positive and was also capable of nitrogen fixation. Moreover, it had maximum swimming (8.9 mm) and swarming (8.7 mm) activities after 24 h, indicating its best PGP traits for future use.

## GRAPHICAL ABSTRACT

**Figure:**
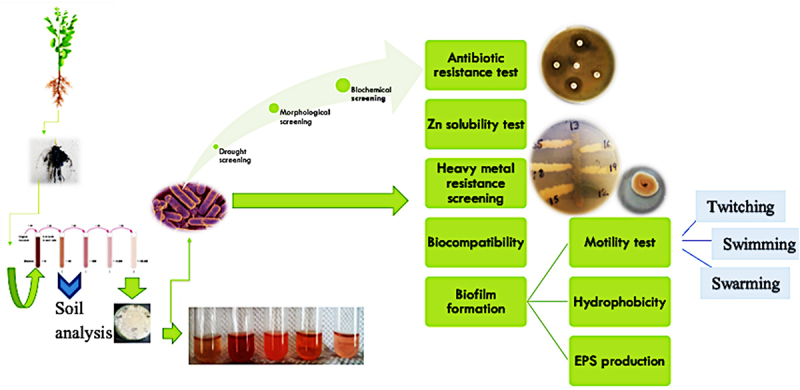

## Introduction

The soil zone under direct effect of plant roots is known as rhizosphere, and the bacteria colonizing plant roots are called Rhizobacteria and only 2–5% of them are plant growth promoting rhizobacteria (PGPR).^1,2^ PGPR is an imperative group of microbial communities because they are reported as growth encouraging and improving micro-nutrients availability to host plants by their special mechanisms and growth promoting secretions.^3,4^ These microbial populations of rhizosphere are comparatively different compared to the surroundings owing to the presence of root exudates, serving as a food source for their survival.^5^ In return, these PGPR play a noteworthy character in enhancing host plant growth in natural plus hazardous circumstances. They promote host plant growth dramatically either by producing growth hormones or by facilitating their nutrient supply from soil.^6^ In actual, these PGPRs behave like bio fertilizers, having no cost, no harm to soil. Moreover, concerns about food security and human survival have emerged as a result of the world’s population growth, economic development, and human activity that is causing soil ecosystems to deteriorate and nonrenewable resources to run out.^6^ Strong ecological awareness has led to a focus on plant growth-promoting rhizobacteria (PGPR) as a solution to these negative effects. Through both direct and indirect methods, PGPR can promote plant growth and increase crop output. Many parts of the world are actively utilizing PGPR’s capacity to enhance soil quality and encourage plant growth in order to increase agricultural productivity.^7^ Utilizing microorganisms offers promising solutions to ensure sustainable agricultural production. Thus, microorganisms play a crucial role in agricultural biomes, especially plant growth-promoting microbes, by actively participating in vital biological processes like nitrogen fixation and phytohormone synthesis, making essential nutrients soluble, as well as serving natural enemies against pests and diseases, they increase crop yields.^8^

In addition, many of these rhizobacteria can also develop host plant tolerance against abiotic and biotic stresses like drought, salinity, flooding and heavy metal stress, and, hence, facilitate plant existence under adverse environmental circumstances.^9^ Among all stresses, drought is the most limiting one, affecting plant growth and its yield. PGPR can directly increase endorsement of micronutrients and affect hormonal homeostasis or indirectly provoke the immune system against phytopathogens.^10^ Plant growth promoting rhizobacteria belong to a wide category of autotrophic/independent soil microbia. However, not all bacteria in a given genus have the same metabolic capacities to promote plant growth to the same degree.^11,12^ Suitable selection and screening measures are imperative to investigate proficient PGP rhizobacteria to use as biofertilizer^13^ and to combat abiotic stresses in addition.

In the labs, various screening techniques have been employed to choose possible candidates. Both direct and indirect strategies are being used. The capacity of rhizobacteria for HCN, ACC deaminase production, biocidal and antagonistic activity against pathogens, and other properties are included in the indirect approach. These attributes of rhizobacteria aid in the promotion of plant growth by boosting plant vigor, root growth, seed germination, and yield.^14–16^ On the other hand, direct or conventional approaches include selection based on the ability to produce phytohormones (ethylene, cytokinin and auxin), phosphorus solubilization and nitrogen fixing activities.^17^

In the current study, an assorted approach was implemented for PGPR collection from different areas and different plants, in order to isolate a large variety of indigenous microflora, which might serve with multitrait characteristics, for plant growth promotion under drought stress.^18^ Rhizobacterial screening has various attributes like ability to tolerate drought, solubilize P and N fixation as well as production of indole acetic acid (IAA). The evaluation of isolated strains was done on the basis of their biochemical and morphological qualities. Moreover, their PGP traits were further quantified by adopting different techniques.

## Materials and methods

### Isolation and purification of rhizobacteria

The soil samples of rhizospheres were obtained from cities of Multan (30°N, 71°E), Khanewal (30.8°N, 71.93°E), Mian chanoo (30.43°N, 72.35°E), Lahore (31.52°N, 74.35°E), Sheikhupura (31.71°N, 73.98°E), Gujrat (32.57°N, 74.1°E) and Faisalabad (30.15°N, 71.52°E) in Punjab province. A total of 50 soil samples (rhizospheric) were collected in sterilized, properly labeled polyethylene bags, placed in ice box and brought to the laboratory for further dispensation.

Soil samples were divided into suspensions and analyses, prepared in autoclaved distilled water, and serial dilutions (10^−1^ to 10^−6^) were made to analyze the suspensions.^2^ Samples were inoculated into LB broth, incubated at 25 ± 2°C for 24 h, diluted and spread (10^−3^) on L-agar plates. Different colonies were observed, selected to streak, and morphology (of pure colonies) recorded.

### Soil analysis

The collected soil samples were analyzed for their physical and chemical properties. For physical properties, soil type (structure and texture) was examined.^19^ For chemical properties, soil samples were submitted to the soil and water testing laboratory, Multan, where conductivity, pH, organic matter, K and P contents were estimated from the samples.

### Screening for auxin production

Isolated strains were screened on the basis of their ability to produce Auxin. Evaluation was done by Calorimetric method, in which, tryptophan amended broth was inoculated with isolated strains and incubated for 72 h. Absorbance of incubated mixtures was checked at 530 nm. A standard curve was drawn from synthetic IAA concentration series for assessment of IAA concentrations formed by isolated bacteria.^20^

### Screening for drought tolerance

Drought tolerance of rhizospheric isolates was examined by growing them on at diverse drought levels. Polyethylene glycol (PEG, 6000) was expended to generate drought stress in YEM broth^21^ as previously described.^18^ The levels expended were 0%, 5% (−0.06 Mpa), 10% (−0.03 Mpa), 15% (−0.10 Mpa) and 20% (−0.14 Mpa) of PEG solution.

### Morphological and biochemical characterization

Isolated drought tolerant strains were characterized on the cradle of their morphological, biochemical characteristics and staining outcomes. The cell and colony morphology was recorded. Moreover, their spore forming abilities were also denoted. Biochemical characterization of isolated strains was done using standard protocols used for production of various compounds, enzymes or metabolites. These tests included carbohydrate fermentation test,^22^ enzymes production (gelatinase, catalase and oxidase) test,^23^ indole test, hydrogen sulfide (H_2_S) production test,^24^ MRVP (methyl red and Proskauer) test. Moreover, isolated strains were screened out for plant growth enhancing attributes like production of auxins,^25^ phosphorus solubilization^26^ nitrogen fixation and HCN production.^27^

Isolated strains were tested for carbohydrate fermentation, producing acid or gas depending on their type. Fermentation broth containing (trypticase, sodium chloride, carbohydrate, Phenol red, distilled water) was prepared, placed in autoclaved tubes, inoculated with fresh cultures, and incubated at 35 ± 2°C for 3–5 d. Gas bubbles were produced to indicate sugar fermentation.

For phosphorus solubilization, NBRIP medium having tricalcium phosphate as insoluble compound was used,^28^ isolated strains were grown as spot inoculation in culture plates. Positive strains were identified on the basis of hollow zone production on media plates. Jensen’s medium^29^ was used to screen isolated strains for their nitrogen fixing ability. The pre-labeled, sterilized medium containing plates were used to spot inoculation with isolated strains for 3 to 7 d (28 ± 2°C). Positive strains for nitrogen fixation were checked for hallow zones. For activity of HCN production, modified agar with 4.4 g of glycine per liter was used and spot inoculation was done. Whatman filter paper, soaked in picrate solution (2% Na2Co_3_, 0.5%picric acid) then dried, was used in lids of media plates. Inverted media plates were incubated for 4–5 d. Color change from yellow to orange brown was observed for positive strains.^30^

### Antibiotic sensitivity/resistance screening

Disk diffusion method was opted for antibiotic sensitivity*/resistance* pattern for each isolated rhizobacteria.^31^ For this, fresh bacterial cultures in broth were incubated for 24 h. A small amount of broth (100 µl) was streaked on individually-labeled agar plates for every strain. On every streak plate, a different antibiotic disc was placed. For 24 h, the plates were incubated at 25°C. Chloramphenicol (C 30), Ciprofloxacin (CIP), Enrofloxacin (Enr 10), Norfloxacin (Nor 10), Amoxicillin (AML 10) and chlortetracycline (CT 10) were the antibiotic discs that were used. After a 24-h incubation period, the presence or absence of inhibition zones, surrounding the antibiotic discs were examined.

### Zinc solubility test

The capacity of each isolate to bind the zinc source was examined by tris minimal medium amended with zinc source.^32^ The colony diameter was used to track the strains’ growth.

### Heavy metal tolerance of bacterial strains

The isolated screened strains were inoculated in LB agar media plates supplemented with 200ug/ml of different heavy metal salts^33^ to check their tolerance ability against these metals. The metal salts used were copper sulfate (Cu), cobalt chloride (Co), ferrous sulfate (Fe), lead acetate (Hg), Potassium permanganate (Mn) and zinc sulfate (Zn).

### Biocompatibility test

Biocompatibility of isolated rhizobacteria was determined. The strains were streaked on L agar plates in such a way that one strain was streaked in the middle as straight line, while other strains streak lines were perpendicular to it. The test plates were incubated at 25°C for 24–48 h. The streak lines intersecting the middle one was considered as compatible to that strain, while that inhibited the growth were incompatible strains.^34^

### Motility tests of screened strains

Colonization behavior of the strains was checked in terms of their motility. The twitching swimming and swarming assays were performed. LB with 1% agar was inoculated by stabbing for twitching. Incubation was done at 25 ± 2°C for 24–48 h. Growth was observed in the form of hazy zones.^35^ Swimming and swarming media plates were spot inoculated, incubated and were observed as migration patterns of bacterial cells away from their inoculation spots. Swimming and swarming zone diameters were calculated.^36,37^

### Screening for hydrophobicity of bacterial strains

Cell surface hydrophobicity of selected strains was checked by following the method of Rosenberg.^38^ The hydrophobicity of the cell surface was measured by measuring the decrease in absorbance of the lower aqueous phase. Bacterial strains were cultured in nutrient broth, and their percent hydrophobicity was calculated by subtracting the absorbance of second and fourth day from absorbance of lower aqueous phase of the first day at 400 nm.^39^

### Screening for exopolysaccharides (EPS) production

The EPS quantification essay was performed as described by Wiencek et al.^40^ The weight of precipitates made of EPS was recorded in µgml^−1^.

### Genetic analysis

The 24 h incubated cultures of 12 selected drought tolerant strains (on the basis of their PGP characteristics) were submitted to Macrogen company, Seoul Korea for genetic analysis. The 16S rRNA gene sequences obtained were submitted to GenBank and accession numbers were obtained. The phylogenetic relations were checked between the selected drought tolerant strains and their neighboring strains by using software MEGA 7.

### Statistical analysis

All tests and trials were conducted thrice at least, with three to five times replication. SPSS (Version 17) was used for Statistical analyses to calculate LSD, and Microsoft Office Excel 2007 was used to calculate means, standard errors and graphics.

## Results

### Isolation and purification of rhizobacteria

**Figure 1.**
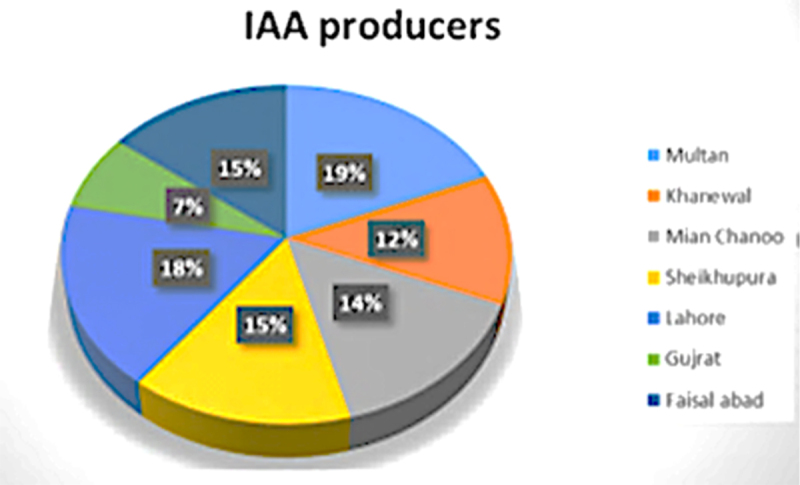
The percentage of auxin-producing strains from different cities of Punjab.

The selected cities of Punjab for sample collection were: Multan denoted by MUX, Khanewal denoted by KW, Mian Chanoo denoted by MCH, Sheikhupura denoted by SKP, Lahore denoted by LHE, Gujrat denoted by GRT, and Faisalabad denoted by LYP. In order to gather a variety of rhizobacteria, the chosen plant categories included trees, cultivated crops, wild plants, and a few ornamental plants. The trees like plants were *Eucalyptus globulus*, *Bombax ceba*, *Ficus carica*, *Mangifera indica*, *Ziziphus jujuba* etc. wild plants were *Chenopodium album*, *Solanum nigrum*, *Acrynthus aspera*, *Cnicus arvensis*, cultivated plants were *Triticum aestivum*, *Psidium guajava*. While ornamental plants were *Hibiscus rosa-sinensis, Rosa indica*, and *Rostenia regia* (Table 1). The rhizobacteria isolated from said regions contributed 19% from MUX, 12% from KW, a 14% was from MCH, 15% after SKP, 18% from LHE, 7% strains from GRT, whereas 15% were from LYP (Figure 1).

**Table 1.** Rhizobacterial screening based on biosynthesis of IAA, tolerance against PEG stress, phosphorus solubilization (PS) and nitrogen fixation (NF).

Plant source	Soil sample	Strains	IAA (ug/ml)	Strains tolerant to PEG stress (20%)	PSB	N fixers
*Mangifera indica*	KW1	M1	115.41±1.5	+	+	+
*Psidium guajava*	KW2	M2	67.41±0.5	+	+	+
*Ziziphus jujube*	KW3	M3	77.56±0.25	+	+	+
*Ziziphus jujube*	KW3	M4	64.14±0.2	+	+	+
*Phoenix dactylifera*	MCH1	M5	88.05±0.25	-	-	-
*Hibiscus rosa-sinensis*	MCH2	M6	45.22±1.5	-	+	-
*Melia azedarach*	MCH3	M7	95.97±0.25	+	-	-
*Melia azedarach*	MCH3	M8	113.17±0.15	-	-	-
*Azadirachta indica*	MCH4	M9	34.72±0.15	-	+	-
*Azadirachta indica*	MCH4	M10	146.54±1.5	-	-	-
*Calotropis procera*	SKP1	M11	93.73±0.2	+	+	+
*Calotropis procera*	SKP1	M12	96.83±0.25	+	+	+
*Eugania jambolina*	SKP2	M13	28.88±0.2	+	+	-
*Cnicus arvensis*	LHE1	M15	115.75±2.5	+	+	-
*Ficus carica*	LHE2	M16	107.49±2	+	-	+
*Dalbergia sissoo*	GRT1	M17	84.1±1.5	-	-	-
*Dalbergia sissoo*	GRT1	M18	85.47±0.25	+	-	-
*Dalbergia sissoo*	GRT1	M19	93.9±0.15	+	-	-
*Dalbergia sissoo*	GRT1	M20	79.8±1.5	+	-	-
*Fabaceous*	GRT2	M21	97.51±0.2	+	+	-
*Eucalyptus globulus*	LYP1	M22	104.40±2	+	+	+
*Eucalyptus globulus*	LYP1	M23	85.64±0.23	+	+	-
*Eucalyptus globulus*	LYP1	M24	63.28±0.1	-	+	-
*Eucalyptus globulus*	LYP1	M25	57.26±0.05	-	+	-
*Ficus religiosa*	LYP2	M26	185.425±2	-	+	+
*Ficus religiosa*	LYP2	M27	110.59±1.5	+	+	+
*Euphorbia helioscopia*	LHE3	M28	378.44±2.5	+	-	+
*Euphorbia helioscopia*	LHE3	M29	105.26±1.5	+	-	+
*Melia azerdarcta*	LYP3	M30	65.17±0.02	+	+	+
*Chenopodium morale*	LHE4	M31	136.39±2.5	-	-	+
*Chenopodium morale*	LHE4	M32	43.50±0.02	-	-	-
*Solanum nigrum*	LHE5	M33	67.24±0.05	-	-	-
*Solanum nigrum*	LHE5	M34	76.53±1.5	-	-	-
*Roystonea regia*	LYP4	M35	85.99±0.2	+	-	+

In order to isolate each strain, the soil dilution method was used. From L-agar plates, distinct colonies were chosen, and they were purified by streaking and restreaking on different agar plates.

### Soil analysis

Analysis of the composition and physical characteristics of every soil sample was conducted. The organic matter content ranged from 0.32% to 0.59%, the pH value varied between 7.5 and 8.9, value of potassium content was from 160 to 210 ppm, and the electrical conductance range was from 2.57 to 10.45 mScm^−1^. Nearly all of the samples were blocky, loamy, and angular or sub-angular (Table 2).

**Table 2.** Soil analysis of collected rhizospheric samples.

Soil sample	pH	O/M %	P(ppm)	K(ppm)	EC mScm^−1^	Structure
1	8±0.15	0.51±0.02	4.26±0.07	192±1	4.38±0.01	Blocky
2	8.3±0.1	0.32±0.01	5.2±0.25	165±2	2.47±0.06	Blocky, sub-angular.
3	8.9±0.2	0.164±0.106	4.3±0.2	172±1.5	2.866±0.015	Blocky, sub-angular
4	8.9±0.2	0.164±0.10	4.3±0.2	172±1.5	2.86±0.015	Sub angular, blocky
5	8.1±0.15	0.51±0.005	4.8±0.2	180.6±1.04	3.91±0.012	Blocky, sub angular,
6	8.3±0.15	0.486667±0.01	4.6±0.2	196±1.5	4.29±0.047	Blocky, sub angular
7	8±0.25	0.56±0.009	4.1±0.15	166.6±3.81	10.45±0.024	Blocky, sub angular
8	8±0.25	0.56±0.009	4.1±0.15	166.66±3.81	10.45±0.024	Sub angular, blocky
9	8.2±0.050	0.42±0.015	4.5±0.15	190±2.5	5.15±0.025	Sub angular, blocky
10	8.2±0.050	0.42±0.0152	4.5±0.15	190±2.5	5.15±0.025	Sub angular, blocky
11	8.4±0.132	0.36±0.015	5.6±0.15	176±2	3.55±0.049	Blocky, sub angular
12	8.2±0.2	0.36±0.015	5.6±0.15	176±2	3.55±0.049	Blocky, sub angular
13	8.7±0.15	0.31±0.017	5.1±0.15	155±1	6.41±0.035	Blocky, sub angular
14	8.7±0.15	0.49±0.02	5.9±0.2	176±2	9.66±0.02	Blocky, sub angular
15	8.3±0.1	0.55±0.02	4.8±0.2	182±1.5	3.80±0.022	Blocky
16	8.1±0.2	0.46±0.02	4.8±0.2	200±2.5	6.25±0.064	Sub angular, blocky
17	8.1±0.2	0.46±0.02	4.8±0.2	200±2.5	6.25±0.064	Sub angular, blocky
18	8.1±0.2	0.46±0.02	4.8±0.2	200±2.5	6.25±0.064	Sub angular, blocky
19	8.1±0.2	0.46±0.02	4.8±0.2	200±2.5	6.25±0.064	Sub angular, blocky
20	8.8±0.1	0.39±0.015	4.7±0.2	206±3.7	7.45±0.024	Blocky, sub angular
21	8.8±0.1	0.39±0.015	4.7±0.2	206.66±3.785	7.45±0.024	Blocky, sub angular
22	8.8±0.1	0.39±0.015	4.7±0.2	206.66±3.785	7.45±0.024	Blocky, sub angular
23	8.8±0.1	0.39±0.015	4.7±0.2	206.66±3.785	7.45±0.024	Blocky, sub angular
24	8.8±0.1	0.39±0.015	4.7±0.2	206±3.78	7.45±0.024	Blocky, sub angular
25	8.2±0.15	0.42±0.02	4.2±0.2	160±1	3.22±0.049	Blocky, loam
26	8.2±0.15	0.42±0.02	4.2±0.2	160±1	3.22±0.049	Blocky
27	8.2±0.15	0.42±0.02	4.2±0.2	160±1	3.22±0.049	Blocky
28	8.3±0.25	0.59±0.02	5.8±0.1	188±1	7.92±0.03	Blocky, sub angular
29	8.3±0.25	0.59±0.02	5.8±0.1	188±1	7.92±0.03	Blocky, sub angular
30	8.6±0.15	0.52±0.02	5.3±0.2	178±1	7.11±0.052	Sub angular, blocky
31	8.6±0.15	0.52±0.02	5.3±0.2	178±1	7.11±0.052	Sub angular, blocky
32	8.53±0.11	0.49±0.02	5.6±0.05	166±1	8.66±0.049	Sub angular, blocky
33	8.5±0.11	0.49±0.02	5.6±0.05	166±1	8.66±0.049	Sub angular, blocky
34	8.3±0.1	0.56±0.015	4.8±0.25	178±1	3.23	Blocky
35	7.5±0.15	0.55±0.01	4.31±0.07	186±0.09	4.5±0.01	Blocky

### Screening for auxin production

The effectiveness of particular strains for IAA production with the addition 1000 µg ml-1 of L tryptophan was assessed using a colorimetric technique. Thirty-four strains produced IAA out of 199 isolated strains. One strain from MUX, four from KW, six from MCH, two from SKP, eight from LHE, five from GRT, and eight from LYP are the strains that produce IAA according to city. The candidates chosen for further action had IAA production in the range of 28.88–378.54 µgml^−1^.

The four bacterial strains from KHW, assigned M1, M2, M3, and M4, generated IAA. M1 yielded the highest amount of auxin, 115.41 µg ml^−1^, when compared to the other three samples. From MCH soil samples, the five strains were acknowledged as M5, M6, M7, M8, M9, and M10. The M9 strain produced the least amount of auxin (34.72 µg ml^−1^) and the M10 strain produced the most (146.54 µg ml^−1^). The strains from SKP were given the designations M11, M12, and. The M14 came from a single MUX sample. Better strains for auxin production were thought to be M13, M15, and M16 of LHE (115.75, 107.49 µg ml^−1^). The four isolates (M17, M18, M19, and M20) from the same GRT sample yielded more and upto 70 µg ml^−1^ of IAA.

An Isolate designated, M21 (yeilded from GRT2), manufactured 97.51 µg ml^−1^ of auxin. The soil sample LYP1 contributed four strains M22, 23, 24 and 25, whereas LYP2 sample added M26, M27 strains having the potential of 185.42, 110.59 µg ml^−1^ IAA production. The strains M28, M29 isolated from LHE3, contributed the maximum auxin as 378.44, 105.26 µg ml^−1^. The two strains designated as M30, M35 isolated from the soil sample LYP3,4, whereas, isolates M31–M34 were extracted from LHE 4 and LHE5 soil samples (Table 2).

### Screening of drought tolerance

The strains that screened positive for auxin production were chosen based on how well they handled drought stress. In order to grow strains, PEG 6000 was used to create drought stress levels in broth of 5%, 10%, 15%, and 20%. The strains which could grow in 20% PEG stress level were considered drought tolerant (60% of the total strains). A total of 21 strains were drought tolerant from 34 IAA producing rhizobacteria. The rhizobacteria M1- M4, M7, M11- M13, M15, M16, M18–M23, M27–M30 and M35 were drought tolerant upto 20% of PEG stress (Table 2).

### Morphological and biochemical characterization

Most strains had circular colonies that ranged in diameter between 1.8 and 3.00 mm. The diameters of the irregular colonies M14, M17, M18, M27, M28, and M35 had from 2.30 to 3.00 mm. The eight colonies had varying shades of green, one had a pink shade (M35), another had a pinkish shade (M18), and the remaining colonies were off-white in color. With the exception of six strains, almost all colonies had entire margins; the outlines of M14 and M27 were lobate, while M17, M18, M28, and M35 had wavy margins. The greenish-colored colonies having grapes or tortilla odor, whereas the white colonies either smelled like tortillas or had no scent at all. Most colonies had elevated surface, while the remaining were relatively flat (Table S1 supplementary file).

The selected strains were all rods most of them were stained as gram negative, capable of capsule formation (M1, M2, M4, M5, M6, M7, M10, M11, M12, M13, M15, M16 and M19), while rest of them were gram positive and had the ability to form spores (M8, M9, M14, M17, M18, M21, M22, M27, M28, M29 and M35). The spores were terminal or central looking as stainless areas by staining with malachite green.

**Figure 2.**
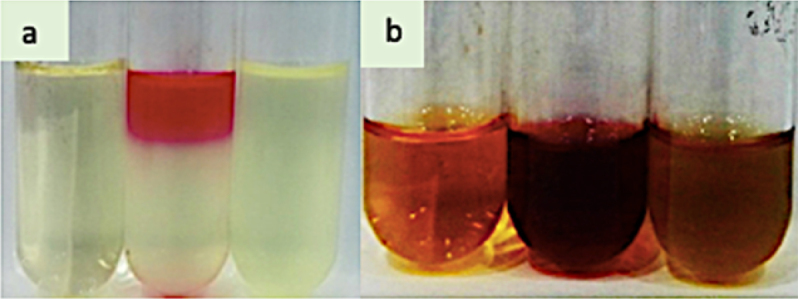
Biochemical characterization a, Indole red test (red colored ring present at top of test tube); b, methyl red test (red color production in culture tube) of isolated drought tolerant rhizobacteria.

The Gram-negative strains also exhibited growth when inoculated on MacConkey agar. Those strains were positive for catalase as well as H_2_S production too. The eight strains (M1, M3, M4, M6, M7, M8, M9 and M35) were capable of producing methyl red, while eight strains (M4, M8, M11, M13, M15, M19, M21, M29) had the ability of indole red formation (Figure 2). Nearly all gram-negative rods produced black precipitates of H_2_S gas. The eight rhizobacterial isolates (M1, M4, M7, M11, M15, M16, M19 and M27) indicated the production of cytochrome oxidase hence were oxidase positive (Table 3).

**Table 3.** Antibiotic sensitivity (zones of inhibition) and resistance (no zones) patterns of isolated drought tolerant rhizobacterial strains.

Strains	Sensitivity/resistance against antibiotics								
	CT 10	N 10	DO 30	AML 10	C 30	CIP 5	S 10	Enr 5	Nor 5
M1	R	R	R	S	R	R	R	R	R
M2	R	S	S	S	S	R	R	R	R
M3	R	R	S	S	S	R	R	R	R
M4	R	R	R	R	R	R	R	R	R
M7	R	R	R	R	R	R	R	R	R
M8	R	R	R	S	R	R	R	R	R
M9	R	R	R	R	R	R	R	R	R
M10	R	R	R	R	R	R	R	R	R
M11	R	R	R	S	R	R	R	R	R
M12	S	R	R	R	R	R	R	R	R
M13	R	S	R	S	R	R	R	R	R
M14	S	R	R	R	R	R	R	R	R
M15	R	S	S	S	S	R	R	R	R
M16	R	R	R	S	R	R	R	R	R
M18	R	S	R	S	S	R	R	R	R
M19	S	R	R	R	R	R	R	R	R
M21	R	R	R	R	R	R	R	R	R
M22	S	R	R	R	R	R	R	R	R
M26	S	R	R	R	R	R	R	R	R
M27	S	R	R	S	R	R	R	R	R
M28	R	R	R	R	R	R	R	R	R
M29	S	R	R	R	R	R	R	R	R
M35	S	R	R	R	R	R	R	R	R

### Antibiotic sensitivity/resistance screening

**Figure 3.**
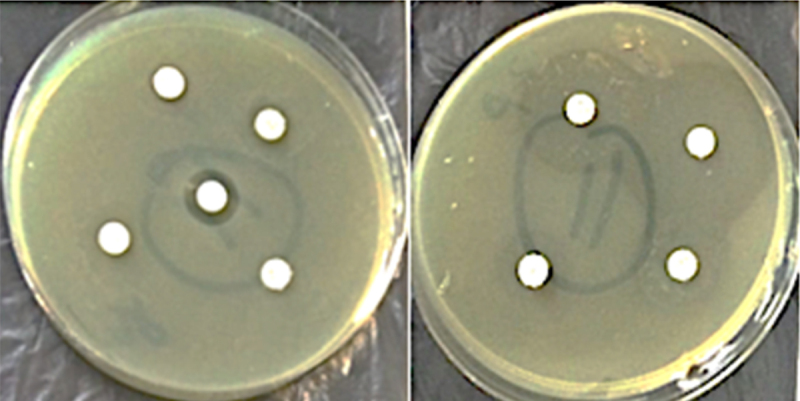
Antibiotic sensitivity and resistance patterns of isolate M11 to different antibiotics.

Every isolated bacterial cell was examined for antibiotic sensitivity or resistance using the disk diffusion method. After a 24-h incubation period, the zones of inhibition surrounding the antibiotic discs were examined (Figure 3). All of the strains exhibited resistance to norfloxacin (Nor 5), enrofloxacin (Enr 5), streptomycin (S 10), and ciprofloxacin (CIP 5). Chlortetracycline (CT 10) was effective against the strains M12, M14, M19, M21, M22, M26, M27, M29, M30, M31, M32, M34, and M35. Chlortetracycline resistance was present in strains M1, M2, M3, M4, M6, M7, M8, M9, M10, M11, M13, M15, M16, M18, M28, M33, and M34. Four antibiotics [neomycin denoted by N 10, doxycycline as DO 30, amoxicillin by AML 10 and chloramphenicol with C 30] were found to be effective against the strains M2 and M15 (Table 3).

### Zinc solubility test and other traits

The capability of screened isolates to dissolve the zinc source in the medium was examined. In this activity, 15 strains (M1, M2, M3, M4, M6–M16, and M18) tested positive, whereas strains M19 through M35 tested negative. The zinc solubilization of strains was validated in terms of solubilization, ranged from 0.4 to 2 cm). In comparison to other strains, the solubility powers of strains M1, M4, M7, M11, and M16 were having high solubilization zones. Their clearing gaps measured over 1 cm (Table 4).

**Table 4.** Biochemical and PGP characterization of stress tolerant rhizobacterial strains by different tests.

*Strains*	*Catalase*	*Gelatinase*	*Oxidase*	*H_2_S*	*Indole*	*Methyl red*	*VP test*	*Zinc solubility zone(cm)*	HCN (ppm)
M1	***+***	***+***	***+***	***+***	-	-	***+***	**1.5±.05**	**26.16± 0.23**
M2	***+***	-	***+***	***+***	-	***+***	***+***	**0.8±.05**	**25.58± 0.19**
M3	***+***	-	***+***	***+***	-	***+***	***+***	**1±.05**	**10.27± 0.18**
M4	***+***	***+***	***+***	-	***+***	-	***+***	**2±.05**	**30.33±0.11**
M5	-	***+***	-	-	-	-	***+***	**0.9±.05**	**9.43±0.07**
M6	-	-	-	-	-	***+***	***+***	**0.8±.07**	**7.4±0.15**
M7	***+***	***+***	***+***	***+***	-	-	***+***	**0.4±.07**	**30.66±0.08**
M8	-	***+***	-	-	***+***	***+***	***+***	**0.45±.005**	**6.766±0.076**
M9	-	-	-	-	-	***+***	***+***	**0.6±.05**	**30.176±0.053**
M10	-	***+***	-	-	-	-	***+***	**1.2±.02**	**9.733±0.104**
M11	***+***	***+***	***+***	***+***	***+***	-	***+***	**0.8±.021**	-
M12	-	***+***	-	-	-	-	***+***	**0.7±.013**	**31.5±0.25**
M13	-	***+***	-	-	***+***	-	***+***	**0.71±.005**	-
M14	-	***+***	-	-	-	-	***+***	-	**13.74±0.115**
M15	***+***	***+***	***+***	***+***	***+***	-	***+***	**1.3±.05**	**8.72±0.101**
M16	***+***	-	***+***	***+***	-	-	***+***	**0.3±.05**	-
M17	-	***+***	-	-	-	-	***+***	-	**5.6±0.049**
M18	-	***+***	-	-	-	-	***+***	-	**10.073±0.05**
M19	***+***	***+***	***+***	***+***	***+***	-	***+***	-	**32.256±0.063**
M21	-	***+***	-	-	***+***	-	***+***	-	-
M22	-	-	-	-	-	-	***+***	-	**40.12±0.052**
M27	-	***+***	***+***	-	-	-	***+***	-	-
M28	-	***+***	-	-	-	-	***+***	-	-
M29	-	***+***	-	-	***+***	-	***+***	-	-
M35	-	-	-	-	-	-	***+***	-	**27.97±0.06**

**Figure 4.**
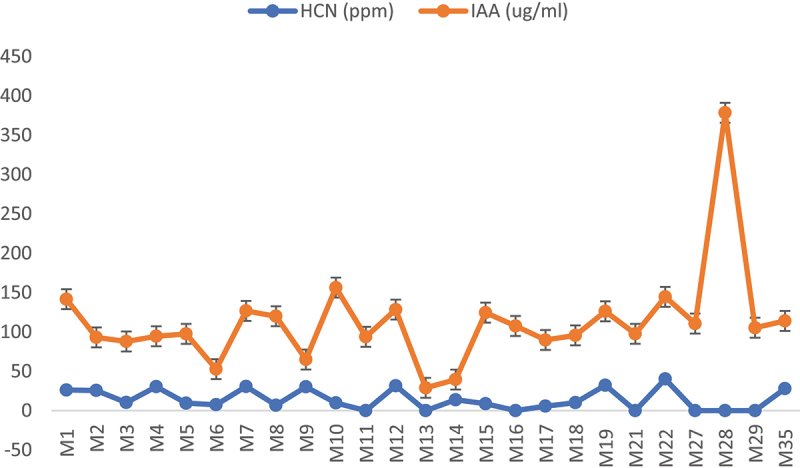
Production of IAA (µg/ml) and HCN (ppm) by isolated strains from different cities of Punjab.

Moreover, comparing different traits of screened bacteria led to diverse scenarios. The strain (M4, from KWN) having significant maximum values of HCN production (30.33ppm) and zinc solubilization (2 cm), may not be proficient to produce maximum IAA, although positive for the said trait. M15 (from LHR) was good in zinc solubilization (1.3 cm) and IAA production (115 µg/ml) but could not yield HCN (ppm). Same was the case with M22 (from LYP) which was capable of IAA production more than 100 µg/ml and HCN 40.12 ppm but was not capable of zinc solubilization (Figure 4, Table 4). In addition, all selected strains, were drought tolerant, IAA producers plus capable of different PGP traits; HCN or zinc solubilization proficient to keep them in PGPR category, hence future candidates for sustainable agriculture.

### Heavy metal tolerance of bacterial strains

The isolated screened strains were inoculated in media plates modified with salts (200 µg ml^−1^) of diverse heavy metal to check their tolerance ability against these metals. The three Strains (M1, M15, M22) were found to be resistant to all used metals except copper. The 11 strains (M2, M3, M6, M8, M10, M12, M13, M14, M29 and M33) exhibited sensitivity to all metals, with no signs of growth on media plates. The two screened strains M19 and M21 showed maximum tolerance to cobalt. M22 expressed tolerance to all heavy metal salts (200 µg ml^−1^) under observation (Table 5). tolerance zone diameters were expressed as + = 0.2–0.7 cm, ++ = 0.8–1.5 cm, +++ = 1.6–2 cm, ++++ = 2.1–2.5 cm.

**Table 5.** Heavy metal resistance activity of rhizospheric, stress tolerant strains isolated from different plants against different metal salts (200ug/ml).

strains	Cu	Co	Fe	Hg	Mn	Zn
M1	-	+	+	+	+	+++
M2	-	-	-	-	-	-
M3	-	-	-	-	-	-
M4	-	+	+	-	+	+++
M6	-	-	-	-	-	-
M7	+	+	+	-	++	+++
M8	-	-	-	-	-	-
M9	-	-	-	-	-	+++
M10	-	-	-	-	-	-
M11	-	+	++	-	+	++
M12	-	-	-	-	-	-
M13	-	-	-	-	-	-
M14	-	-	-	-	-	-
M15	-	+++	+	+++	+	+
M16	-	+	++	-	++	+++
M18	-	-	-	-	-	-
M19	-	++++	+++	-	+++	-
M20	-	-	-	-	-	-
M21	-	++++	+++	-	+++	++
M22	++	++	++	+++	+++	++
M27	-	+++	+	-	+++	+
M28	-	++	+++	-	+++	+
M29	-	-	-	-	-	-
M35	-	+++	+++	+	++	+++

### Biocompatibility test

**Figure 5.**
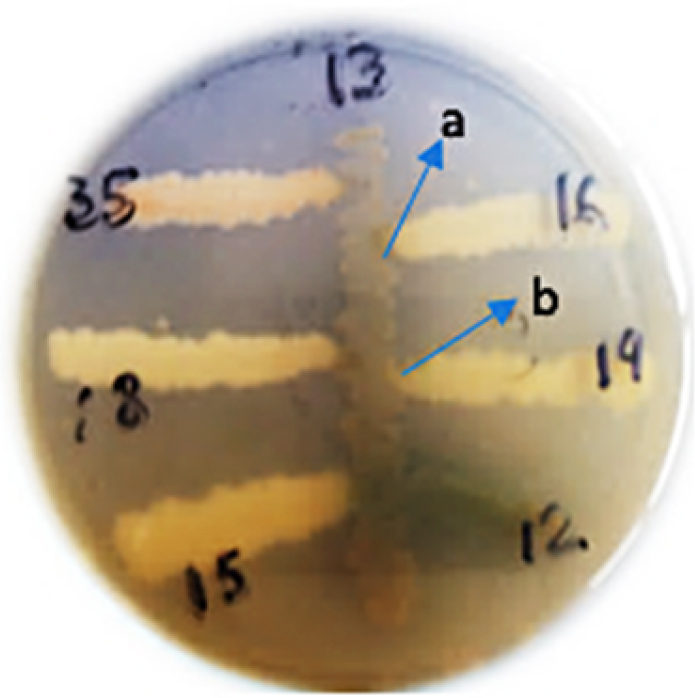
Biocompatibility patterns of isolated rhizobacteria, (a) zone of incompatibility, (b) compatible zone.

The chosen drought-tolerant strains were screened for their biocompatibility with one another, by streaking one strain through the middle of agar plate and others perpendicular to central one (Figure 5). The strain M1 was incompatible with M2 and showed compatibility with M7, M9, M13, M12 and M16 while strain M21 was compatible with strains M1, M2, M11, M13, M16 and M18. Inhibition zones were developed between M13 and M35, M16, M18, M15, while it showed biocompatibility with strain M12 and M19. Moreover, M22 showed compatibility with M27, M28, M31, M29 and M32; although it was incompatible with M30. The strain M30 was incompatible with M22. Biocompatible strains can be grown together as consortium to inoculate plants as bio fertilizer.

### Motility test of screened strains

Motility test was performed in terms of swimming, swarming and twitching using three kinds of agar plates (with varying concentrations of agar) with stab method. The patterns of twitching were seen as hallow zones, while swimming and swarming patterns observed as zone sizes.

**Figure 6.**
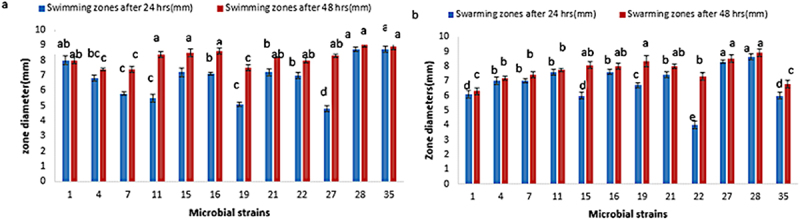
(a) swimming and (b) swarming zones of different rhizobacterial strains, expressed in mm, lettering at bars denote significance (by DMRT), same letters express no significance between and among the groups.

After 24 and 48 h of incubation, the maximum (8.7 mm) swimming zone of strains M28 and M35 was 9 mm and 8.9 mm, respectively. While the swarming activity of M28 (again) had the highest zone diameter of 8.9 mm, and 48 h later, M27 had 8.5 mm. After a full day, the least amount of motility was exhibited by M27 (4.8 mm) and M22 (4 mm), respectively (Figure 6).

### Screening for hydrophobicity of bacterial strains

**Figure 7.**
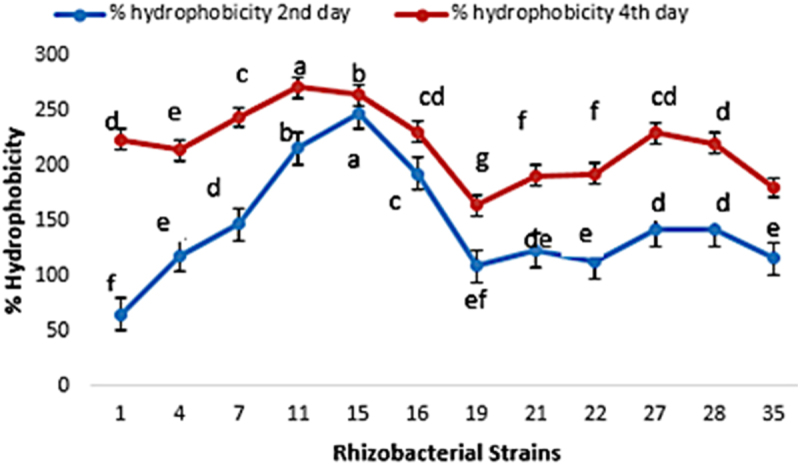
Percent hydrophobicity of selected rhizobacteria (M1, M4, M7, M11, M15, M16, M19, M21, M22, M27, M28 and M35) after 2nd and 4th day of inoculation in growth medium.

Isolated strains expressed diverse conduct of adhesion to hydrocarbons as well as percent hydrophobicity (Figure 7). The absorbance of first, second and fourth day of bacterial broth cultures at 400 nm was recorded, and the percent hydrophobicity was calculated by subtracting second- and fourth-day absorbance from the absorbance of the first day. The isolate M15 exhibited maximum percent hydrophobicity as 248% and 65% on the second day. After 4 d, that hydrophobicity was at 271% by strain M11, exhibiting more hydrophobic behavior as compared to others, followed by M15 that was 264%. The strain M35 showed least hydrophobicity as 180%.

### Screening for EPS production of bacterial strains

**Figure 8.**
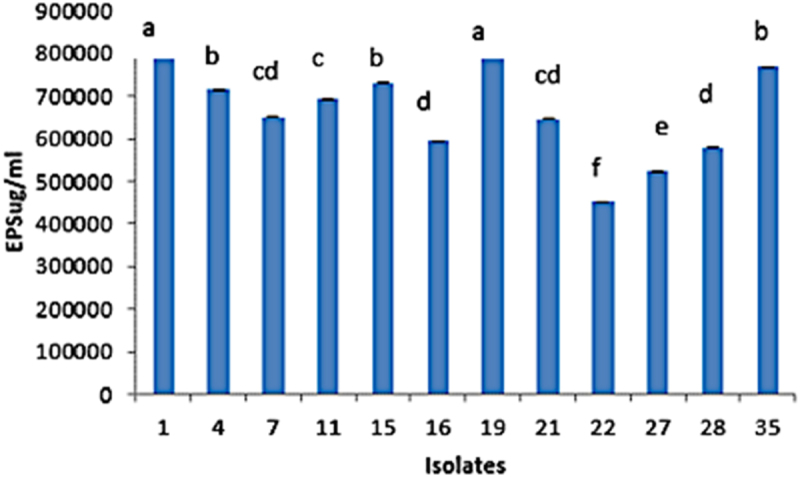
Biofilm production in ug/ml of EPS in selected microbial rhizobacteria (M1, M4, M7, M11, M15, M16, M19, M21, M22, M27, M28 and M35).

The biofilm formation in terms of exopolysaccharides was discovered to be highest in strain M1 (805067 ± 0.002 µg/ml) and M19 (805534 ± 0.002 µg/ml). The least production was shown by strain M22 as 451,034 ± 0.002 µg ml^−1^ (Figure 8).

### Genetic analysis

**Figure 9.**
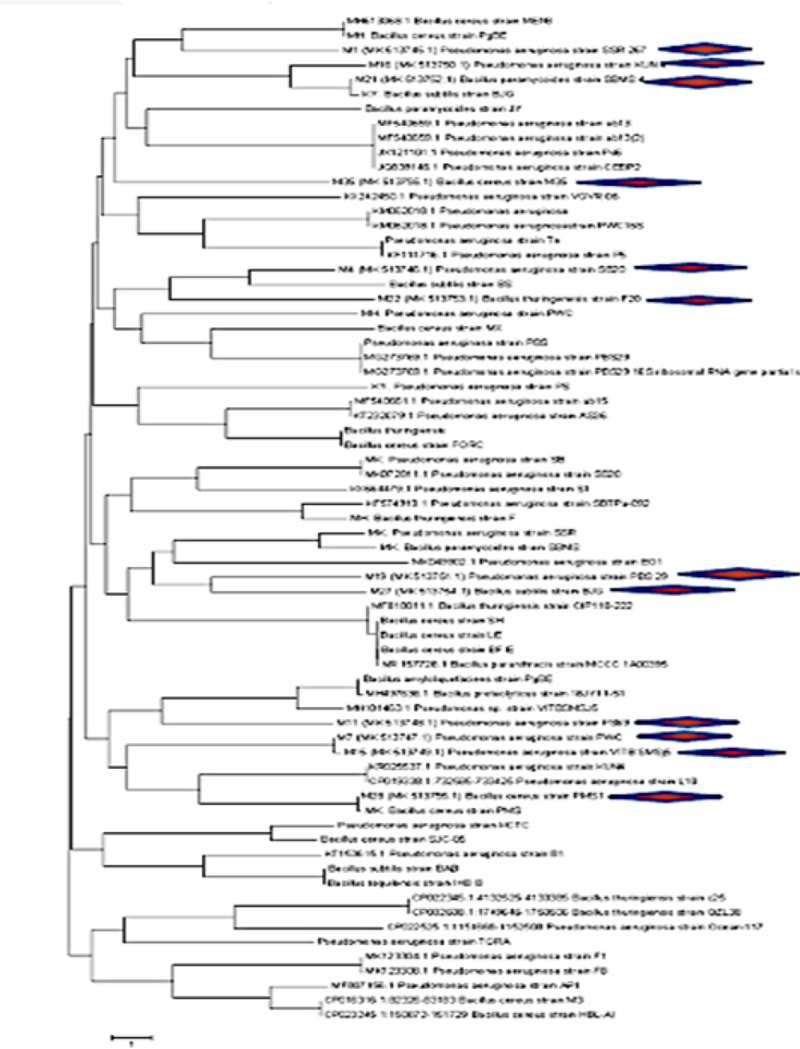
Phylogenetic relationships among isolated drought tolerant strains and their respective neighbor strains on the basis of ribotyping, recovered from GenBank, with the neighbor joining method (the software used, MEGA7). Scale bar displays two nucleotide substitutions for 100 nucleotides.

The accession numbers of 12 selected isolates (M1, M4, M7, M11, M15, M16, M19, M21, M22, M27, M28 AND M35) were MK 513,745, MK 513,746, MK 513,747, MK 513,748, MK 513,749, MK 513,750, MK 513,751, MK 513,752, MK 513,753, MK 513,754, MK 5,137,455 and MK 513,756. The strain MK 513,745, MK 513,746, MK 513,747, MK 513,748, MK 513,749, MK 513,750 and MK 513,751 identified as different strains of *Pseudomonas aeruginosa* (Proteobacteria), while MK 513,752, MK 513,753, MK 513,754, MK 5,137,455 and MK 513,756 were belonged to Genus *Bacillus*, belonging to firmicutes (Table 6; Figures S1, S2, S3 supplementary file). The evolutionary tree was drawn to scale, with branch lengths in the same units as those of the evolutionary distances used to infer the phylogenetic tree. The evolutionary distances were measured in base substitutions per site and were calculated using the Maximum Composite Likelihood method (Figure 9). All isolated, characterized strains were previously identified, by other researchers and no novel strain was identified. Most of the strains were reported as plant associated (as PGPR) by other scientists.

**Table 6.** Genetic documentation of drought tolerant rhizospheric strains isolated from diverse plants.

Isolates	Plant source	Sequence length	% homology	Identified as	Accession number
M1	*Mangifera indica*	1501	100	*Pseudomonas aeruginosa* strain SSR267	Mk513745.1
M4	*Ziziphus jujube*	1501	100	*Pseudomonas aeruginosa* strain SB20	Mk513746.1
M7	*Melia azedarach*	1441	100	*Pseudomonas aeruginosa* strain PWC	Mk513747.1
M11	*Calotropis procera*	1441	100	*Pseudomonas aeruginosa* strain PS69	Mk513748.1
M15	*Cnicus arvensis*	1501	100	*Pseudomonas aeruginosa* strain VITB SMSJ5	Mk513749.1
M16	*Ficus carica*	1441	100	*Pseudomonas aeruginosa* strain KUN 4	Mk513750.1
M19	*Dalbergia sissoo*	1501	100	*Pseudomonas aeruginosa* strain PBS 29	Mk513751.1
M21	*Fabaceous*	1501	100	*Bacillus parmycoides* SBM S4	Mk513752.1
M22	*Eucalyptus globulus*	1561	100	*Bacillus thuringiensis* strain *F 20*	Mk513753.1
M27	*Ficus religiosa*	1561	100	*Bacillus subtilis* strain BJG	Mk513754.1
M28	*Euphorbia helioscopia*	1501	100	*Bacillus cereus* strain PMS1	Mk513755.1
M35	*Roystonea regia*	1441	100	*Bacillus cereus* strain M35	Mk513756.1

## Discussion

Selecting viable candidates (rhizobacteria) that exhibit plant growth-promoting properties is a fundamental and crucial step toward enhancing agricultural practices. This process also offers a substitute fertilizer source as well.^41,42^ In addition to improving plant growth, the screening data may aid in the selection of the most promising candidates to address the scientific challenge. Isolating rhizobacteria with multitrait PGP activities and drought tolerance capacity was the primary goal of the current investigation. For this reason, seven drought prone locations (in Multan, Faisalabad, Gujrat, Mian Chanoo, Khanewal, Sheikhupura, and Lahore) in Punjab were selected. Out of 51 collected soil rhizospheric samples, 199 rhizobacteria were isolated. In the process of screening, the total bacterial strains purified were 28% from soil samples of Multan, 16% strains of Khanewal, 21% of Mian Chanoo, 10% of Sheikhupura, 13% of Lahore, 3% of Gujrat, while 9% were of Faisalabad soil samples and 34 strains were IAA positive. Khalid et al.^43^; Prasad and Raghuwanshi^44^; Jochum et al.^45^; Mazumdar et al.^46^ and Ali & Pati^47^ also isolated and screened PGPR with PGP attributes. During the screening process, 18 strains from Multan, 12 were from Khanewal, 14 obtained from Mian Chanoo, while 14 were from Sheikhupura, 17 obtained from Lahore, 7 were from Gujrat, and 14 from Faisal Abad were among the 96 strains that were detected IAA positive by producing pink coloration in Salkowski reagent test (Calorimetric method). The strains which were positive for auxin production were then screened for drought stress tolerance by using different levels of PEG stress. PEG 6000 is used to induce water stress, does not enter cell wall spaces, and its grains having molecular weight more than 3000.^48^ Kumari et al.^49^ and Niu *et al*.^30^ also screened their strains like this method. They used three PGPR out of nine; four strains, respectively, that were found to be stress tolerant, which were later characterized biochemically. In this screening session, 30 stress-resistant bacteria with the capacity to produce IAA were chosen. A morphological characterization was done on the selected isolates. Most strains had round, entire, raised as well as flat colonies that were mostly off-white in color. Others had lobates, round, entire, or greenish appearances, and they smelled like tortillas or grapes. Gram staining is used to differentiate members of Eubacteriacea into two basic groups, gram positive and gram negative, on the basis of attaining crystal violet iodine complex. Most of the strains were violet colored rods (gram positive) with endospores positioning central or terminal looking oval, non-stained areas by malachite green, while rest of the isolates were pinkish rods (gram negative) with attribute of capsule formation.

The strain M1, M4, M7, M11, M15, M16 and M19 presented large plain colonies on MacConkey agar, positive in H_2_S gas production, Catalase, oxidase positive. The H_2_S gas produced by bacteria is lipophilic, permeable to biological membranes, guards them not only from antibiotics but also counteracts oxidative stress also. It acts as an antioxidant buffer.^50,51^ The catalase test is to differentiate between aerobes and anaerobes. The Catalase positive bacteria are strictly aerobes,^52^ catalase production is helpful to alleviate oxidative stress of H_2_O_2_.

The 15 strains exhibited zinc solubilization characteristics (M1, M2, M3, M4, M6, M7, M8, M9, M10, M11, M12, M13, M14, M16 and M18) while remaining strain (M19 to M35) were negative for the activity. In comparison to other strains, the solubility powers of strains M1, M4, M7, M11, and M16 were high. One additional benefit of some PGPR that helps with the biofortification of staple crops is the solubilization of insoluble zinc compounds.^53^ Inoculums containing strains capable of solubilizing zinc can enhance soil conditions, promote plant growth, and increase productivity in zinc-deficient soils. The same strategy was applied to promote wheat growth by Sirohi et al.^54^ In the heavy metal screening test, with the exception of copper salt, strains M1 and M22 demonstrated tolerance to every metal salt tested (200 ug/ml). All metals exhibited sensitivity in the strains M2, M3, M6, M8, M10, M12, M13, M14, M29, and M33. Most of all, the M19 and M21 showed tolerance to cobalt salt. According to Meliani and Bensoltane,^55^ biofilm formation in rhizobacteria that are resistant to heavy metals helps them maintain a favorable niche in stressful environments, particularly in soil that has been contaminated with metals. Metal-resistant isolates were utilized by Verma et al.,^56^ Patel et al.,^57^ and Pramanik et al.^58^ to promote the growth of wheat, peanuts, and rice.

The strains’ biocompatibility behavior was inconsistent. Between the junctions of two incompatible strains was the zone of inhibition. Bacterial consortia are biocompatible species that can be used as co-inoculants to promote plant growth.^59^ Incompatible strains impede the promotion of growth by halting the formation of biofilms.^60^ The ability of isolated strains to form biofilms was tested. The communities of bacteria plus their extracellular polymeric secretions (EPS) combine to form biofilms. Different characteristics such as the ability to swim and swarm, hydrophobicity of the cell surface, and EPS production are either directly or indirectly linked to the formation of biofilms. According to Ansari and Ahmad,^61^ biofilms play a role in the synthesis of inhibitory compounds and resistance to abiotic stresses.

Every rhizobacterium exhibited positive motility, exhibiting patterns of colony formation through activities such as twitching, swimming, and swarming. According to Boelens et al.,^62^ the motility of bacterial strains is crucial for their ability to clone in roots and soil as it affects their survival and even distribution in the soil for colonization. Swarming in *Bacillus subtilis* was suggested by Gao et al.^63^ as a migration strategy for tomato plant root colonization.

The bacterial colonization of the host root surface is dependent on the hydrophobicity and charge of the cell surface.^64^ Extracellular polymers are responsible for this colonization or adhesion; persistent bacteria will only stay firmly attached if the bacterial surface is cleaned with salt solutions, water, or buffers.^65^ *Pseudomonas tolaasii* has been observed to attach itself to plant roots due to the presence of bivalent ions of calcium, strontium, and magnesium.^66^ Due to their negative charge, these cations make it easier for bacteria to stick to root surfaces.^67^

Production of extracellular polymer (EPS) improves bacterial endurance, antibody resistance, antimicrobial compound production, and rhizosphere colonization.^68^ According to Zheng et al.,^69^ rhizobacteria that can produce EPS slow down evaporation and increase the amount of water available to plants, giving them more time to adapt to water stress.

The best strains were chosen based on their ability to withstand drought and their biochemical characteristics. These strains were genetically identified, and the Neighbor-Joining method was used to infer their evolutionary history. Within the Proteobacteria and Firmicutes taxa, the strains were grouped into two groups: the predominant genus, Pseudomonas, and the genera Bacillus. In order to promote plant growth, Niu et al.,^30^ Jochum et al. (2019), and Danish et al.^70^ also screened out *Pseudomonas and Bacillus* spp. and other drought-tolerant bacteria. Bacteria that are being isolated from plant rhizospheres and display PGP traits such as zinc solubility, IAA and HCN production, antibiotic resistance, and efficiency in motility and biofilm formation can be employed for crop production and yield vigor.^46^ The isolates M1, M4, M7, M11, M15, M16, and M19 in the current investigation, are the best potential candidates for improving plant growth in a sustainable agriculture system.

## Conclusion

The present study suggests that strains isolated from plant rhizospheres with PGP characteristics, such as Auxin production, zinc solubility, HCN production, efficiency in motility and biofilm formation along with drought tolerance, can promote plant growth and are promising aspirants of sustainable agriculture.

## Supplementary Material

supplementary data Isolation.docx

## Data Availability

All data is presented in the article and raw data is available on request.
