# Supplementary material for: Exploring the efficacy of drought tolerant, IAA-producing plant growth-promoting rhizobacteria for sustainable agriculture
Source: Plant Signal Behav. 2025 Jan 15;20(1):2452331. doi: 10.1080/15592324.2025.2452331 (PMC11740683; doi:10.1080/15592324.2025.2452331)
Supplement: supplementary data Isolation.docx [file KPSB_A_2452331_SM6982.docx]

**Table 1**. Colony and cell morphology of drought stress tolerant isolated strains

| Strains | **Colony morphology** | | | | **Cell morphology** | | | | |
| --- | --- | --- | --- | --- | --- | --- | --- | --- | --- |
|  | **Shape, size(mm)** | **Color** | **Margin** | **Elevation** | **Odor** | **Shape** | **Gram staining** | **Spore staining** | **Capsule staining** |
| M1 | **Round, 1.8** | **Green** | **Entire** | **Flat** | **Grape like** | **rod** | **-ve** | **-** | **+** |
| M2 | **Round, 1.8** | **Green** | **Entire** | **Flat** | **Tortilla** | **rod** | **-ve** | **-** | **+** |
| M3 | **Round, 1.9** | **Green** | **Entire** | **Raised** | **tortilla** | **rod** | **-ve** | **-** | **+** |
| M4 | **Round, 1.9** | **Green** | **Entire** | **Raised** | **Grape** | **rod** | **-ve** | **-** | **+** |
| M5 | **Round, 2** | **Greenish** | **Entire** | **Raised** | **Grape** | **rod** | **-ve** | **-** | **+** |
| M6 | **Round, 2.1** | **Off white** | **Entire** | **Flat** | **-** | **rod** | **-ve** | **-** | **+** |
| M7 | **Round, 2** | **Greenish** | **Entire** | **Raised** | **Grape** | **rod** | **-ve** | **-** | **+** |
| M10 | **Round, 2.9** | **Off white** | **Entire** | **Raised** | **-** | **rod** | **-ve** | **-** | **+** |
| M11 | **Round, 2.1** | **Off white** | **Entire** | **Flat** | **tortilla** | **rod** | **-ve** | **-** | **+** |
| M12 | **Round,2.7** | **White** | **Entire** | **Raised** | **-** | **rod** | **-ve** | **-** | **+** |
| M13 | **Round, 2.3** | **White** | **Entire** | **Raised** | **-** | **rod** | **-ve** | **-** | **+** |
| M15 | **Round, 2.5** | **Greenish** | **Entire** | **Flat** | **Grape** | **rod** | **-ve** | **-** | **+** |
| M16 | **Round, 2.3** | **Off white** | **Entire** | **Flat** | **tortilla** | **rod** | **-ve** | **-** | **+** |
| M19 | **Round, 2.9** | **Off white** | **Entire** | **Raised** | **grape** | **rod** | **-ve** | **-** | **+** |
| M8 | **Round, 2.5** | **Greenish** | **Entire** | **Flat** | **-** | **rod** | **+ve** | **+** | **-** |
| M9 | **Round, 2.3** | **Off white** | **Entire** | **Flat** | **-** | **rod** | **+ve** | **+** | **-** |
| M14 | **irregular, 3** | **White** | **Lobate** | **Flat** | **-** | **rod** | **+ve** | **+** | **-** |
| M17 | **Irregular** | **Off white** | **Wavy** | **Raised** | **-** | **rod** | **+ve** | **+** | **-** |
| M18 | **Irregular, large** | **Pinkish** | **Wavy** | **Raised** | **-** | **rod** | **+ve** | **+** | **-** |
| M21 | **Round,2.7** | **White** | **Entire** | **Raised** | **-** | **rod** | **+ve** | **+** | **-** |
| M22 | **Round, 2.3** | **White** | **Entire** | **Raised** | **-** | **rod** | **+ve** | **+** | **-** |
| M27 | **irregular, 3** | **White** | **Lobate** | **Flat** | **-** | **rod** | **+ve** | **+** | **-** |
| M28 | **Irregular,** | **Off white** | **Wavy** | **Raised** | **-** | **rod** | **+ve** | **+** | **-** |
| M29 | **Round,** | **Off white** | **Entire** | **Flat** | **-** | **rod** | **+ve** | **+** | **-** |
| M35 | **Irregular, large** | **Pink** | **Wavy** | **Raised** | **-** | **rod** | **+ve** | **+** | **-** |


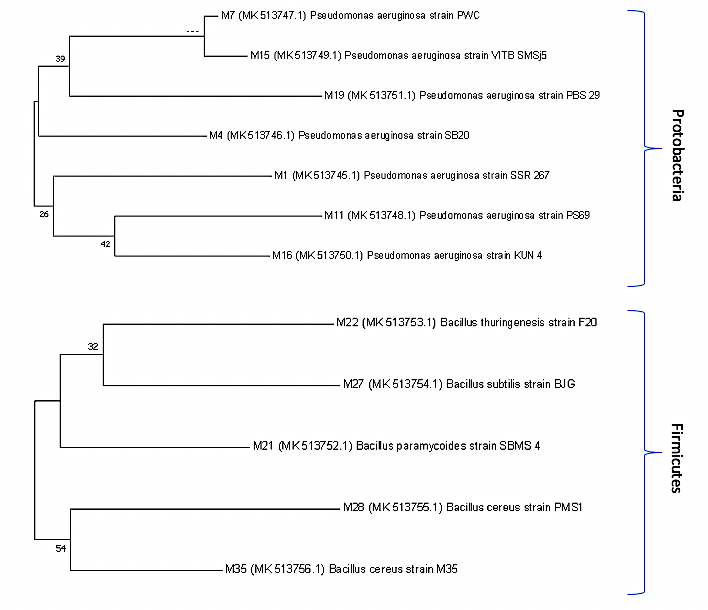


**Figure 1: Taxonomic evolutionary relationship of two groups of isolated drought tolerant rhizobacteria, as clustered in bootstrap test using MEGA 7.**


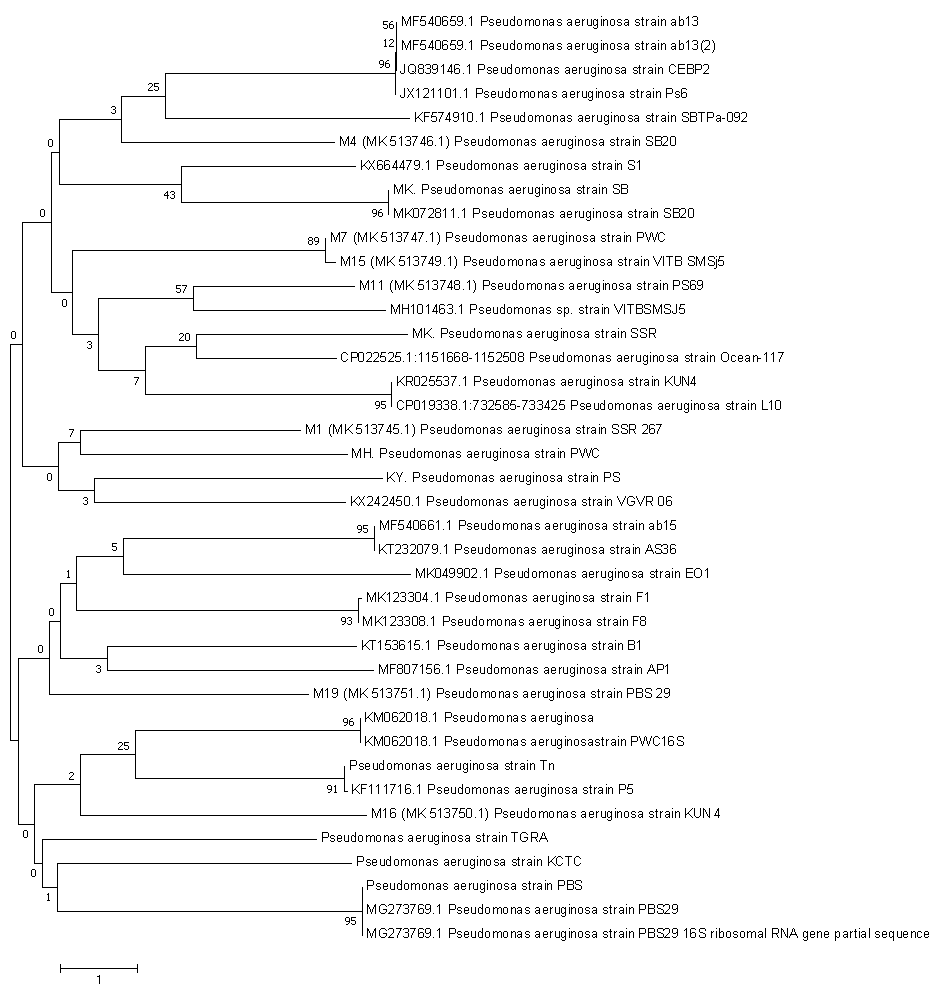


**Figure 2: Evolutionary relationships of isolated drought tolerant *Pseudomonas aeruginosa* strains with their neighbor strains**


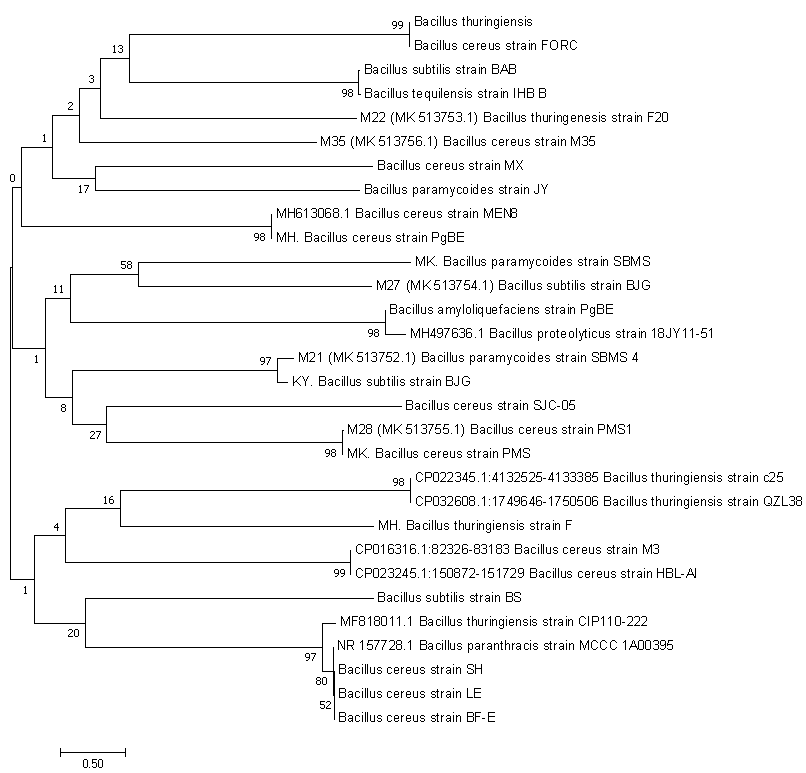


**Figure 3: Evolutionary relationships of isolated drought tolerant Bacilli with their neighbor strains**
